# Assessment of hyperactivity-impulsivity and attention deficit in adolescents by self-report and its association with psychopathology and academic performance

**DOI:** 10.3389/fpsyg.2022.989610

**Published:** 2022-08-18

**Authors:** Pedro Saura-Garre, Jose L. Vicente-Escudero, Silvia Checa, Maravillas Castro, Visitación Fernández, Mavi Alcántara, Antonia Martínez, Concepción López-Soler

**Affiliations:** Faculty of Psychology, University of Murcia, Murcia, Spain

**Keywords:** ADHD, self-report, adolescents, psychopathology, academic performance

## Abstract

The scientific literature highlights the risk of the appearance of internalizing and externalizing symptoms, together with difficulties in the academic area, linked to diagnosis of Attention Deficit Hyperactivity Disorder (ADHD). This is normally assessed by teachers and primary caregivers, disregarding the self-perception of the adolescents themselves, which limits detection of this disorder at an evolutionary stage. Our aim was to analyze the psychometric properties of a self-report for ADHD in adolescence and its relationship with psychopathology and academic performance. This study assessed an incidental sample of 267 students from secondary schools in the Region of Murcia, Spain, using the EDAH questionnaire adapted for self-report, in order to analyze its psychometric properties in assessing ADHD. The Youth Self-Report (YSR) and the Brief Self-Control Scale (BSCS) were also used to determine their association with psychopathological, self-control and academic performance variables. An ADHD prevalence of between 3.7 and 13.1% was observed depending on the established cut-off point. The adapted EDAH showed adequate reliability indices (α = 0.818; ω = 0.817) and explained a high variance percentage (50.655%). Adolescents with anxiety/depression difficulties, dissocial behavior, aggressiveness, and poor performance in mathematics showed a higher amount of ADHD symptoms. Moreover, self-control, dissocial behavior, age, and performance in Social Sciences acted as predictors of the disorder. The good psychometric properties of this questionnaire and its adequate correspondence with other variables of interest suggest it is an appropriate self-report instrument to assess ADHD in adolescence.

## Introduction

Several studies show prevalence’s of Attention Deficit Hyperactivity Disorder (ADHD) ranges between 8.8 and 10–12% in the general population of adolescents aged between 11 and 19 years old (Ghossoub et al., 2017; Danielson et al., 2018; Umar et al., 2018), higher than the 5% suggested by the DSM-5 (American Psychiatric Association, 2013).

As regards ADHD diagnosis, Mhalla et al. (2018) found factors linked to its appearance, such as poor academic performance, negative parental relationship, family history of psychopathological problems, and the presence of somatization, sleep disorders and addictive behaviors (the Internet, videogames, alcohol, tobacco, and cannabis). Among these, Internet abuse [Odds Ratio (*OR*) = 2.39] and a negative parental relationships (*OR* = 16.43) appear as possible risk factors for onset of ADHD, as well as a personal history of psychopathological problems (*OR* = 12.16) and exposure to abuse (*OR* = 3.16). Martin (2014) finds that ADHD significantly predicts low academic performance (*OR* = 2.21), change of school (*OR* = 1.78), and expulsion rate (*OR* = 6.98). Tsai et al. (2017) found as risk factors in the child-adolescent population the lack of prosocial behaviors (*OR* = 0.87 in the inattentive subtype; *OR* = 0.68 hyperactive subtype) and poor school performance (*OR* = 1.23 in the inattentive subtype; *OR* = 1.55 in the hyperactive subtype). In this study, ADHD was also significantly linked to greater externalizing problems and lower family adjustment. Research by Kuja-Halkola et al. (2015) describes the relationship between externalizing symptomatology and ADHD. These authors found a significant influence between the presence of externalizing behaviors in childhood and the appearance of ADHD traits at the start of adolescence. Moreover, the relationship between both variables appears to increase over time. As for internalizing symptoms, Sevincok et al. (2020) indicate a prevalence of 4.79% of anxious-depressive symptomatology, 2.72% of withdrawal and 1% of somatic complaints among adolescents with ADHD.

For ADHD assessment and diagnosis, various scales have been developed to detect symptoms and/or inattentive behaviors and hyperactivity/impulsivity, as well as structured interviews based on DSM diagnostic criteria. This detection is normally by observers close to the children and adolescents, mainly teachers and parents or principal caregivers, as these best understand the minors habitual behavior pattern (Güler et al., 2017). Data provided by both parents and teachers on this type of behavior are equally valid (Bied et al., 2017), along with presenting differential characteristics which show the importance of using both informants for more reliable diagnosis (Narad et al., 2015), but it is known that externalizing disorders (such as ADHD) are more visible in the family and school context involving higher rates of clinical diagnoses, but a good diagnosis is not made or is more complicated when referring to internalizing problems in children, with only information from external informants (López-Soler et al., 2010). Nevertheless, the use of self-report to learn minors’ perception of their own symptoms does not appear to have been generalized in clinical and research contexts, when there might be significant data to complete diagnosis and subsequent treatment. There has been controversy around ADHD due to doubts created by an increase in diagnosed cases in recent years. A new systematic review with over 300 articles evidenced this over diagnosis in children and adolescents, with the implications of the pharmacological and psychological treatments applied, as well as stigmatization which accompanies this diagnostic label (Kazda et al., 2021).

With this scenario, it seems reasonable to consider the number and nature of informants who can participate in the ADHD diagnostic process, valuing the inclusion of minors’ self-perception as an element which can broaden professional’s vision during assessment. San Nicolás and Iraurgi (2016), developed a self-report scale for boys and girls between 9 and 17 years of age (EA-ADHD) obtaining adequate first results in discrimination between probable and negative ADHD cases. In the case of the adolescent population, the value of using questionnaires designed for adults has also been shown, such as the “ADHD Self-Report Scale (ASRS)” developed by the World Health Organization (Kessler et al., 2005) and validated for children between 13 and 17 years by Adler et al. (2012).

One of the most widely used instruments in Spain is the *”Scale for assessment of attention deficit hyperactivity disorder, EDAH”* (Farré and Narbona, 2003), adapted from the Conners Scale (Conners, 1989), with 20 items assessing the presence of inattentive behaviors, hyperactivity/impulsivity, and behavioral problems observed by teachers. There is a reduced adaptation of the EDAH scale which enables minors themselves, in this case adolescents, to evaluate their behavior supporting information provided by teachers (Álvarez-García et al., 2012). This adaptation showed moderate reliability indices in initial exploratory analyzes. It is necessary however to delve further into this questionnaire’s structure, internal and external validity, and its relationship with variables of psychopathology, self-control, neglect, and academic performance, as these authors only related the questionnaire to aggression within classrooms and schools, showing moderate correlations between ADHD symptoms with physical violence, social exclusion, and disruptive behaviors.

The possible use of this adaptation of the EDAH scale is particularly interesting, as it is a widely known questionnaire used by ADHD diagnostic services and teams in Spain, both at clinical, educational, and research level. Having a simple and reliable adaptation would allow an increase in data obtained in evaluation processes. It would also enable adjustment of diagnosis of the disorder, counting on the participation of the adolescents themselves, as these are who will receive subsequent intervention.

## Materials and methods

### Participants

Incidental cluster sampling was carried out in two secondary schools in the San Javier municipality, Region of Murcia, Spain.

A sample of *n* = 267 students (54.5% female) was selected between the 1st and 4th ESO Compulsory Secondary/High School Education courses. Ages ranged between 12 and 18 years due to the presence of repeating students. The mean (*M*) age of the sample was 14.18 years, with a standard deviation (*SD*) of 1.497. Distribution by sex and by course of the sample is presented (Table 1).

**TABLE 1:** Prevalence by course and sex.

Course	Boys (*n*)	Girls (*n*)	Total (*n*)	*X* ^2^
1°	17.8 (47)	12.5 (33)	30.3 (80)	
2°	9.1 (24)	17 (45)	26.1 (69)	
3°	11.4 (30)	12.1 (32)	23.5 (62)	
4°	7.2 (19)	12.9 (34)	20.1 (53)	11.061; *p* < 0.05; η^2^ = 0.04

To calculate the coefficient η^2^, the course was taken as a reference as an independent variable.

In this sample 20.5% were foreign nationals and 12% claimed to suffer from some illness. 20.2% had failed the Spanish Language and Literature subject, 25% subjects related to Social Sciences and History, 27.75% Mathematics, 26.1% Science subjects such as Physics and Chemistry or Technology, and 15.1% stating they had failed other subjects like Physical Education, Art or Music.

### Instruments

Students were assessed with three questionnaires which measured ADHD, psychopathological problems, academic performance, and self-control, as follows:

*Assessment of adapted Attention Deficit Hyperactivity Disorder* (EDAH). The original EDAH (Farré and Narbona, 2003) is a 20-item instrument on a Likert-type scale with four response options, which identifies presence of the main ADHD symptoms in children between 6 and 12 years of age such as hyperactivity, inattention and concomitant behavioral disorders. This questionnaire is completed by the teacher and has high reliability (α = 0.95). However, the adapted EDAH questionnaire (Álvarez-García et al., 2012) was used in this research which is a self-report of 10 items on a Likert-type scale with four response options (0 not at all; 1 a little; 2 quite; and 3 a lot) for minors between 12 and 18 years, composed of hyperactivity and inattention scale items from the original EDAH. The authors found two factors in the factorization of this questionnaire which explained 49.57% of total variance, these being hyperactivity-impulsivity (α = 0.726) and attention deficit (α = 0.720). The hyperactivity-impulsivity scale comprised the following items: 1 “I am excessively restless”; 8 “I move constantly, I am restless”; 9 “I’m impulsive”; 3 “I frequently annoy my colleagues”; and 5 “I demand immediate satisfaction of my demands.” The attention deficit scale comprised items: 2 “I have learning difficulties at school”; 6 “I am often in the clouds”; 4 “I am easily distracted”; 7 “I leave some tasks unfinished”; and 10 “I am restless, I get frustrated easily.” The total reliability of the questionnaire was high (α = 0.808).

The Spanish adaptation (Lemos et al., 1992) of the Youth Self-Report (YSR) was used, a questionnaire for young people and adolescents aged between 11 and 18 years (Achenbach et al., 1987). The report contains two parts. Firstly, a sociodemographic survey gathering information on sex, age, nationality, school year, and school performance by subject, among other variables. This first part obtained good reliability in its adaptation to Spanish, (α = 0.62). The second part has 112 items on a Likert-type scale with three response options (0 not true; 1 sometimes true; and 2 very often true). This part of the questionnaire assesses emotional and behavioral problems. Two correction scales were used, the Achenbach System of Empirically Based Assessment (ASEBA), which measures internalizing syndromes (anxiety/depression, withdrawal/depression, and somatic complaints), externalizing syndromes (both dissocial and aggressive behavior) and social, thought, and attention problems. Additionally, the DSM problem-oriented scale was also used, which measures affective, anxiety and somatic problems, oppositional defiance, and behavioral problems. This latter part showed high reliability in adaptation to Spanish (α = 0.90).

Brief Self-Control Scale (BSCS) by Tangney et al. (2004) in its Spanish adaptation (Archer et al., 2010), has adequate psychometric properties (α = 0.83). It is a self-report questionnaire answered on a Likert-type scale with five response options ranging from 1 “totally disagree” to 5 “totally agree.” The questionnaire comprises 13 items and a single factor measuring the student’s self-control.

### Procedure

The University of Murcia approved this study. In addition, permission from the school headteachers and written informed consent from the parents of the children was required.

After requesting permission from school headteachers, informed consents were provided to the parents of the minors.

Participants who had received provided signed consent from parents or legal guardians of the child took part in research within 15 days. The groups were given questionnaires in the presence of the researcher. No semantic clarification was provided on items.

Students provided all data voluntarily; confidentiality and anonymity were guaranteed by assigning a code to each participant. The time invested per class in application of questionnaires was around 30°min.

### Data analysis

Exploratory factorial analysis of the adapted EDAH questionnaire was carried out using the principal component extraction method with varimax rotation, as it offered higher communalities and more satisfactory factor loadings. Two factors were extracted. Choice of number of factors was based on three criteria: (1) Analysis of sedimentation graph; (2) The first two factors explained over half of total variance and were the only ones which obtained eigenvalues greater than 1; (3) This decision was based on previous theoretical research (Álvarez-García et al., 2012). The correlation matrix was analyzed with Bartlett’s Sphericity test, the sample adequacy measure using the KMO index, and scale reliability with Cronbach’s *alpha* and McDonald’s *omega* coefficient.

Descriptive statistics of new factors and normality of distribution of scale scores were analyzed. Differences in scores of scales according to gender and course were analyzed.

Percentiles, median and interquartile range were calculated by courses and for the total sample. Cut-off points were calculated, with the 90th percentile as an indicator of moderate risk of ADHD and the 95th percentile as high risk, as shown in the EDAH questionnaire manual (17). Prevalences were calculated based on these cut-off points.

As a measure of external validity, correlations between the new adapted EDAH scales and the empirical and DSM-oriented scales of the YSR questionnaire were analyzed together with the correlation between this questionnaire and the self-control one.

A multiple linear regression model was conducted to determine the possible effect of the YSR psychopathological variables (empirical scales only), academic performance, and self-control on the total ADHD symptom score.

A binary logistic regression model was finally calculated to analyze which psychopathological, self-control, and school performance variables are associated as either a risk or protective factor with ADHD symptoms measured with the adapted EDAH questionnaire.

All analyzes were performed using the IBM SPSS V25 for Windows program.

## Results

Prior to factorial analysis, interdependence of variables was analyzed using Bartlett’s test of Sphericity, determining the interrelation of variables (*X*^2^ = 777.273; gl = 45; *p* < 0.001). The adequacy of the sample was also analyzed and a high KMO index was observed (KMO = 0.829). The two factors, with eigenvalues greater than 1, explained 50.655% of total variance. Inclusion criteria for an item in each factor were that its factorial load was above 30 (Table 2).

**TABLE 2:** Factor loads, communalities, explained variance, eigenvalues, and reliability of the adapted EDAH questionnaire.

	Communality	Factor	
		
		1	2
8. I move constantly, I am restless	0.690	0.852	
1. I am excessively restless	0.399	0.820	
9. I am impulsive	0.380	0.729	
10. I am fickle, I get frustrated easily	0.543	0.595	
5. I demand immediate satisfaction of my demands	0.148	0.300	
7. I leave some tasks unfinished	0.552		0.764
6. I am often in the clouds	0.587		0.702
4. I am easily distracted	0.768		0.675
2. I have school learning difficulties	0.604		0.608
3. I frequently annoy my colleagues	0.395		0.543
Eigenvalues		3.886	1.179
% Explained variance		26.386	24.269
% Cumulative explained variance		26.386	50.655
Cronbach’s *Alpha*		0.751	0.736
McDonald’s *Omega*		0.758	0.733

The first factor explained 26.386% of variance. As for content, this factor analyzes hyperactivity-impulsivity problems and comprised items 8, 1, 9, 10, and 5 with adequate reliability (α = 0.751; ω = 0.758). The second factor explained 24.269% of variance. Regarding content, this factor analyzes attention deficit problems and comprised items 7, 6, 4, 2, and 3, with adequate reliability (α = 0.736; ω = 0.733). Total reliability of the questionnaire was high (α = 0.818; ω = 0.817).

The Kolmogorov-Smirnov test determined that the new scales of the EDAH questionnaire adapted for attention deficit (*Z* = 0.158; gl = 267; *p* < 0.001), hyperactivity-impulsivity (*Z* = 0.112; gl = 267; *p* < 0.001) and total scale (*Z* = 0.118; df = 267; *p* < 0.001) did not follow normal distribution, therefore the non-parametric Kruskal-Wallis test was used in order to analyze differences regarding gender and academic course of these scales (Table 3).

**TABLE 3:** Median and interquartile range of adapted EDAH scales and differences according to gender and course.

		Hyperactivity-impulsivity			Attention deficit			Total scale		
					
		*Mdn* ^1^	*IQR* ^2^	*X*^2^ (*p)*	*Mdn*	*IQR*	*X*^2^ (*p)*	*Mdn*	*IQR*	*X*^2^ (*p)*
**Sex**										
	Boy	4	5	0.027 (0.870)	4	4	1.56 (0.212)	8	8	0.483 (0.487)
	Girl	4	5.75		4	4		9	8.75	
**Course**										
	1° y 2°	4	4	17.62 (< 0.001)	4	5	2.26 (0.133)	7	8	11.91 (< 0.001)
	3° y 4°	5	5		4	4		10	9	
Total		4.81	3.44		4.79	2.91		9.61	5.58	

^1^Median.

^2^Interquartile range.

Academic courses were in two groups, 1st and 2nd of Compulsory Secondary Education in one and 3rd and 4th in the other, since statistically significant differences were found between both groups in all scales, except for attention deficit. No significant differences were found based on gender for any adapted EDAH scale.

The amplitude range of scores for Attention Deficit and Hyperactivity-Impulsivity scales was from 0 to 15 and 0 to 30 for the total scale, although in sample scores from this study, the minimum and maximum range was between 0 and 24 for the total scale, 0 to 15 for the Hyperactivity-Impulsivity scale, and 0 to 13 for the Attention Deficit scale. To calculate cut-off points, scores equal to or greater than the 90th percentile were considered a moderate risk of having ADHD symptoms, and those equal to or greater than the 95th percentile as high risk. Prevalences for these new cut-off points were also calculated (Table 4).

**TABLE 4:** Moderate risk and high risk cut-off points of the adapted EDAH and prevalence for total sample and ESO courses.

		Cut-off points hyperactivity-impulsivity (%)	Cut-off points attention deficit (%)	Cut-off points total scale (%)
Total				
	Cop 90	10 (4.9)	9 (6.4)	17 (8.2)
	Cop 95	12 (4.9)	11 (4.9)	20 (4.5)
1° and 2°				
	Cop 90	9 (2.2)	9 (4)	16 (3.7)
	Cop 95	10 (1.5)	11 (9.4)	20 (1.5)
3° and 4°				
	Cop 90	11 (2.6)	9 (5.3)	19 (4.5)
	Cop 95	12 (3.4)	11 (4.3)	22 (3)

Cut-off points represent the score a subject must obtain on the scale to be considered without risk, moderate risk or high risk.

As an external validity element of the adapted EDAH questionnaire, Spearman’s correlations (ρ) were calculated with the Brief Self-Control questionnaire and the empirical Achenbach and DSM-oriented scales of the YSR questionnaire. Among the adapted EDAH scales, high correlations were seen with the total scale (ρ = 0.894 for Hyperactivity-Impulsivity and ρ = 0.847 for Attention Deficit) and moderate ones between the Hyperactivity-Impulsivity scale and Attention Deficit (ρ = 0.535). Significant correlations were observed in all questionnaires, ranging from ρ = 0.162 minimum to ρ = 0.732 maximum. Scales which obtained best correlations were the Total EDAH Scale (with self-control ρ = −0.623; attention problems ρ = 0.699; externalizing syndromes ρ = 0.645; and DSM attention problems ρ = 0.732), Hyperactivity-Impulsivity (with self-control ρ = −0.511; Attention problems ρ = 0.527; Externalizing syndromes ρ = 0.545; and DSM attention problems ρ = 0.602) and Attention Deficit (with self-control ρ = −0.609; Attention problems ρ = 0.709, externalizing syndromes ρ = 0.576, and attention problems DSM ρ = 0.683) (Table 5).

**TABLE 5:** Correlations between EDAH scales adapted with the self-control questionnaire and youth self-report (YSR) questionnaire.

	Total EDAH scale	Hyperactivity-impulsivity	Attention deficit
Total EDAH scale	1	–	–
Hyperactivity-impulsivity	0.894**	1	–
Attention deficit	0.847**	0.535**	1
Self control	−0.623**	−0.511**	−0.609**
Anxiety/depression	0.314**	0.375**	0.162**
Withdrawal/depression	0.256**	0.209**	0.234**
Somatic complaints	0.258**	0.258**	0.189**
Social problems	0.353**	0.322**	0.299**
Thought problems	0.448**	0.446**	0.329**
Attention problems	0.699**	0.527**	0.709**
Dissocial	0.584**	0.473**	0.548**
Aggressive behaviors	0.580**	0.507**	0.503**
Internalizing	0.323**	0.334**	0.220**
Externalizing	0.645**	0.545**	0.576**
Affective problems DSM	0.397**	0.383**	0.299**
Anxiety problems DSM	0.335**	0.368**	0.218**
Somatic problems DSM	0.248**	0.230**	0.199**
Attention problems DSM	0.732**	0.602**	0.683**
Oppositional defiance DSM	0.451**	0.381**	0.392**
Conduct problems DSM	0.534**	0.398**	0.545**

**p < 0.001.

A multiple linear regression model was calculated to determine the possible effect of the psychopathological variables of the empirical YSR scales, school performance, age, and self-control on students’ total ADHD scores. Since the full-scale variable of the adapted EDAH was not normally distributed which is a requirement to perform a linear regression model, scores were standardized to perform said model. Variables which were significant predictors of ADHD were self-control, antisocial behavior, age, school performance in Social Sciences and History, and anxiety/depression (Table 6).

**TABLE 6:** Multiple regression analysis on the total scale of adapted EDAH.

	B (ET)	*t*	*p*
Constant	3.749	6.908	0.000
Self-control	−0.044 (0.006)	−7.4	0.000
Dissocial conduct	0.047 (0.012)	3.854	0.000
Age	0.108 (0.030)	3.595	0.000
Performance in social sciences	−0.255 (0.095)	−2.693	0.008
Anxiety/depression	0.023 (0.009)	2.602	0.01
*R*^2^ (%)	22.1		
Model	*F* (7; 282) = 13.362; *p* < 0.001		

B, Standardized regression coefficient; ET, Typical error.

To analyze the risk/protection variables most associated with ADHD symptoms, a binary logistic regression model was performed using the “forward” method. To do so, the dependent variables of Attention Deficit, Hyperactivity-Impulsivity and the total adapted EDAH scale were categorized into two treatment levels, considering two ranges, one without significant symptoms, grouping scores below the 90th percentile and another with significant symptomatology, grouping scores equal to or greater than the 90th percentile. Psychopathological problems from the empirical Achenbach scales and school performance were entered as association variables. Self-control was not included in analysis, as it was so well-associated with ADHD scores as a protective variable (for the Hyperactivity-Impulsivity scale Odds Ratio (*OR*) = 0.834; and *OR* = 0.863 for the Attention Deficit scale) that it eliminated the rest of the variables of the model (Table 7).

**TABLE 7:** Attention deficit hyperactivity disorder binary logistic regression analysis adapted with psychopathology and academic performance variables.

	Variables	Exp B (IC 95%)	*Wald*	*p*
**Total scale**				
	Constant	0.003	49.744	0.000
	Anxiety/depression	1.386 (1.167–1.646)	13.816	0.000
	Withdrawal/depression	0.784 (0.623–0.988)	4.254	0.039
	Somatic complaints	0.778 (0.622–0.972)	4.886	0.027
	Dissocial conduct	1.211 (1.052–1.393)	7.141	0.008
	Aggressive conduct	1.172 (1.017–1.35)	4.814	0.028
	Negative performance in mathematics	3.375 (1.233–10.954)	5.457	0.019
Model			104.529	0.000
*R*^2^ (%)		45.9		
**Hyperactivity-impulsivity**				
	Constant	0.017	57.776	0.000
	Thought problems	1.157 (1.022–1.309)	5.347	0.021
	Aggressive conduct	1.138 (1.023–1.265)	5.701	0.017
Model			105.185	0.000
*R*^2^ (%)		20.8		
**Attention deficit**				
	Constant	0.004	55.777	0.000
	Anxiety/depression	1.125 (1.022–1.238)	5.799	0.016
	Dissocial conduct	1.256 (1.129–1.398)	17.564	0.000
	Negative performance in social sciences	6.988 (2.443–19.988)	13.145	0.000
Model			105.739	0.000
*R*^2^ (%)		41.5		

R^2^ was calculated from Nagelkerke.

## Discussion

Attention deficit hyperactivity disorder continues to be over diagnosed in minors, therefore contributing to stigmatization of the disorder as well as application of unjustified pharmacological and psychological treatments (Kazda et al., 2021), therefore instruments must be developed to provide more information to the diagnostic process at different ages.

In our study, the exploratory factor analysis solution resulted in extraction of two factors explaining a high percentage of variance and which have adequate internal consistency, both for the factors and general scale encompassing these. Factor I, Hyperactivity-Impulsivity is which explains most variance.

Unlike other studies, our factor analysis differed in two items. In our research, item 10 “I am inconstant, I get frustrated easily” is within the Hyperactivity-Impulsivity factor, and item 3 “I often annoy my colleagues” is within the Attention Deficit factor. In other research (Farré and Narbona, 2003; Álvarez-García et al., 2012), item 10 is within the latter factor, and item 3 the former. This change in saturation of items in factors may be due to characteristics of the sample object of this study, since ADHD expression characteristics vary throughout the various stages of an individual’s life (Agnew-Blais et al., 2016; Lee et al., 2021).

Different cut-off points were calculated for the questionnaire scales, the total sample and differentiating by academic years. Prevalences obtained in this study range between 2.2 and 8.2% for mild-moderate ADHD symptoms and 1.5–4.9% for severe ADHD symptoms. Combining these two prevalence ranges shows that ADHD of any severity, lies between 3.7 and 13.1%. These results agree with other research data (Ghossoub et al., 2017; Danielson et al., 2018; Umar et al., 2018), that prevalence of ADHD ranges between 8.8 and 12%. This appears to show that the instrument developed in this research might be a reliable tool in measuring ADHD symptoms, performing adequate screening in the school population at the Secondary Education stage.

Moreover, there is concordance between our ADHD scales and externalizing, attention, and self-control problems. These are common in studies examining ADHD and its correlations, and these same relationships are found in other studies (Kuja-Halkola et al., 2015; Vicente-Escudero et al., 2019), indicating that adapted ADHD scales are adequate to measure this problem.

It is observed that self-control (*B* = −0.044), dissocial behavior (*B* = 0.047), age (*B* = 0.108), academic performance in Social Sciences (*B* = −0.255), and anxiety/depression symptoms (*B* = 0.023), are significant predictors of ADHD symptoms. Furthermore, anxiety/depression problems (*OR* = 1,386), presenting dissocial behavior (*OR* = 1,211), aggressive behavior (*OR* = 1,172), inadequate performance in mathematics (*OR* = 3,375), not showing symptoms of withdrawal/depression (*OR* = 0.784), and not manifesting somatic complaints (*OR* = 0.778) are risk factors linked with a greater number of ADHD symptoms. In addition, manifesting thought problems (*OR* = 1.157) and aggressive behaviors (*OR* = 1.138) are risk factors associated with a greater number of Hyperactivity-Impulsivity symptoms. Moreover, symptoms of anxiety/depression (*OR* = 1.125), dissocial behavior (*OR* = 1.256) and inadequate performance in subjects related to Social Sciences (*OR* = 6.988), are more probability with a greater number of attention deficit symptoms. These results coincide with those from other research, that poor academic performance, psychopathological problems, somatizations, sleep disorders, and behaviors related to poor self-control, such as internet abuse, video games or alcohol (Martin, 2014; Mhalla et al., 2018; Vicente-Escudero et al., 2019), are factors significantly linked to the appearance of ADHD symptoms.

These data expand extensive research related to ADHD and provide psychometric validation of a questionnaire administered in self-report format, and how this relates to variables of academic performance, self-control and externalizing, and internalizing problems. This questionnaire can be applied to minors at the secondary educational stage, one in which these questionnaires are quite scarce (Narad et al., 2015; Bied et al., 2017), thus providing an additional measurement that can help prevent over diagnosis of the disorder. Adequate instruments are essential to measure ADHD at different ages, as long-term studies show that children with ADHD continue to present symptoms and comorbid psychological problems during adolescence and early adulthood (Miranda et al., 2015); this being very important since the early detection of this phenomenon would make possible an adequate intervention, because as we find in the scientific literature, the optimal window of opportunity to improve the outcomes of mental disorders is prevention or early treatment (Fusar-Poli, 2019).

This study contains some limitations, particularly in incidental sampling, which focused on only two secondary schools in San Javier, which may limit replicability and generalization of research results. A further limitation is that there are no data from informants other than the minors themselves, such as from teachers or parents, to assess agreement between these three types of informants.

In future research, it would be convenient to assess how this questionnaire relates to other variables of interest, such as its relationship with interaction with parents and relatives of minors, since other research shows significant results in this area (Mhalla et al., 2018). It is also advisable to evaluate minors’ self-concept and self-esteem, since these appear to be relevant variables which influence school performance (Rodríguez-Rodríguez and Guzmán, 2021).

In conclusion, adequate psychometric reliability data, high percentage of variance explained by questionnaire factors, and its relationship and consistency with data found by other researchers all suggest that application of this questionnaire, following this distribution of items and cut-off points, is a reliable and valid measure to evaluate symptoms of total ADHD, Hyperactivity-Impulsivity, and Attention Deficit in minors enrolled in the Compulsory Secondary Education stage.

## Data availability statement

The raw data supporting the conclusions of this article will be made available by the authors, without undue reservation.

## Ethics statement

The studies involving human participants were reviewed and approved by University of Murcia. Written informed consent to participate in this study was provided by the participants or their legal guardian/next of kin.

## Author contributions

PS-G, JV-E, SC, and CL-S have contributed to the conceptualization, formulation and evolution of the research goals and objectives and to the administration, and coordination of the research activity. PS-G, VF, and MA have contributed to the curation and formal analysis of the research data. PS-G, JV-E, and SC contributed to the collection of the research data. SC, MC, and AM were responsible for the provision of research resources and methodology development. JV-E, SC, and CL-S were responsible for writing the initial draft of the manuscript. All authors have contributed substantially to the development, drafting, and final approval of the version to be published.

## References

[B1] Achenbach T. M. Edelbrock C. Howell C. T. (1987). Empirically based assessment of the behavioral/emotional problems of 2- and 3- year-old children. *J. Abnorm. Child. Psychol.* 15 629–650. 10.1007/BF00917246 3437096

[B2] Adler L. A. Shaw D. M. Spencer T. J. Newcorn J. H. Hammerness P. Sitt D. J. (2012). Preliminary examination of the reliability and concurrent validity of the attention-deficit/hyperactivity disorder self-report scale v1.1 symptom checklist to rate symptoms of attention-deficit/hyperactivity disorder in adolescents. *J. Child. Adolesc. Psychopharmacol.* 22 238–244. 10.1089/cap.2011.0062 22537184

[B3] Agnew-Blais J. C. Polanczyk G. Danese A. Wertz J. Moffitt T. E. Arseneault L. (2016). Persistence, remission and emergence of ADHD in young adulthood: Results from a longitudinal, prospective population-based cohort. *JAMA Psychiat.* 73 713–720. 10.1001/jamapsychiatry.2016.0465 27192174 PMC5475268

[B4] Álvarez-García D. Hevia S. M. González-Castro P. Pérez C. R. (2012). Hiperactividad-impulsividad y déficit de atención como predictores de participación en situaciones de violencia escolar. *Int. J. Psychol.* 12 185–202.

[B5] American Psychiatric Association (2013). *Diagnostic and statistical manual of mental disorders*, 5th Edn. Arlington: American Psychiatric Publishing.

[B6] Archer J. Fernández-Fuertes A. A. Thanzami V. L. (2010). Does cost-benefit analysis or self-control predict involvement in two forms of aggression? *Aggress. Behav.* 36 292–304. 10.1002/ab.20358 20665710

[B7] Bied A. Biederman J. Faraone S. (2017). Parent-based diagnosis of ADHD is as accurate as a teacher-based diagnosis of ADHD. *Postgrad. Med.* 129 375–381. 10.1080/00325481.2017.1288064 28271921 PMC5743217

[B8] Conners C. K. (1989). *Manual for Conners’ Rating Scales Toronto.* Canada: Multi-Health Systems Inc.

[B9] Danielson M. L. Bitsko R. H. Ghandour R. M. Holbrook J. R. Kogan M. D. Blumberg S. J. (2018). Prevalence of parent-reported ADHD diagnosis and associated treatment among U.S. children and adolescents, 2016. *J. Clin. Child. Adolesc. Psychol.* 47 199–212. 10.1080/15374416.2017.1417860 29363986 PMC5834391

[B10] Farré A. Narbona J. (2003). *Evaluación del trastorno por déficit de atención con hiperactividad.* Madrid: TEA.

[B11] Fusar-Poli P. (2019). Integrated mental health services for the developmental period (0 to 25 years): A critical review of the evidence. *Front. Psychiat.* 10:355. 10.3389/fpsyt.2019.00355 31231250 PMC6567858

[B12] Ghossoub E. Ghandour L. A. Halabi F. Zeinoun P. Shehab A. A. S. Maalouf F. T. (2017). Prevalence and correlates of ADHD among adolescents in a Beirut community sample: Results from the BEI-PSY Study. *Child. Adolesc. Psychiat. Ment. Health* 11:20. 10.1186/s13034-017-0156-5 28428817 PMC5393010

[B13] Güler A. S. Scahill L. Jeon S. Taşkın B. Dedeoğlu C. Ünal S. (2017). Use of multiple informants to identify children at high risk for ADHD in Turkish school-age children. *J. Atten. Disord.* 21 764–775. 10.1177/1087054714530556 24799319

[B14] Kazda L. Bell K. Thomas R. McGeechan K. Sims R. Barratt A. (2021). Overdiagnosis of attention-deficit/hyperactivity disorder in children and adolescents: A systematic scoping review. *JAMA Netw. Open* 4:e215335. 10.1001/jamanetworkopen.2021.5335 33843998 PMC8042533

[B15] Kessler R. C. Adler L. Ames M. Demler O. Faraone S. Hiripi E. (2005). The World Health Organization Adult ADHD Self-Report Scale (ASRS): A short screening scale for use in the general population. *Psychol. Med.* 35 245–256. 10.1017/s0033291704002892 15841682

[B16] Kuja-Halkola R. Lichtenstein P. D’Onofrio B. M. Larsson H. (2015). Codevelopment of ADHD and externalizing behavior from childhood to adulthood. *J. Child. Psychol. Psychiat.* 56 640–647. 10.1111/jcpp.12340 25303006 PMC4393334

[B17] Lee S.-M. Cheong H.-K. Oh I.-H. Hong M. (2021). Comparison of persistence and adherence between adults diagnosed with attention deficit/hyperactivity disorder in childhood and adulthood. *Neuropsychiatr. Dis. Treat.* 17 3137–3146. 10.2147/NDT.S337819 34703234 PMC8526951

[B18] Lemos S. Fidalgo A. M. Calvo P. Menéndez P. (1992). Salud mental de los adolescentes asturianos. *Psicothema* 4 21–48.

[B19] López-Soler C. Alcántara M. V. Fernández V. Castro M. López Pina J. A. (2010). Características y prevalencia de los problemas de ansiedad, depresión y quejas somáticas en una muestra clínica infantil de 8 a 12 años, mediante el CBCL (Child BehaviorChecklist). *An. de Psicol* 26 325–334.

[B20] Martin A. J. (2014). The role of ADHD in academic adversity: Disentangling ADHD effects from other personal and contextual factors. *Sch. Psychol. Q.* 29 395–408. 10.1037/spq0000069 24820011

[B21] Mhalla A. Guedria A. Brahem T. Amamou B. Sboui W. Gaddour N. (2018). ADHD in tunisian adolescents: Prevalence and associated factors. *J. Atten. Disord.* 22 154–162. 10.1177/1087054717702217 28381094

[B22] Miranda A. Colomer C. Fernández M. I. Presentación M. J. Roselló B. (2015). Analysis of personal and family factors in the persistence of attention deficit hyperactivity disorder: Results of a prospective follow-up study in childhood. *PLoS One* 10:e0128325. 10.1371/journal.pone.0128325 26024216 PMC4449179

[B23] Narad M. E. Garner A. A. Peugh J. L. Tamm L. Antonini T. N. Kingery K. M. (2015). Parent-teacher agreement on ADHD symptoms across development. *Psychol. Assess.* 27 239–248. 10.1037/a0037864 25222436 PMC4495952

[B24] Rodríguez-Rodríguez D. Guzmán R. (2021). Academic performance of secondary education students in socio-familial risk contexts. *Suma. Psicol.* 28 104–111. 10.14349/sumapsi.2021.v28.n2.5 31013238

[B25] San Nicolás S. Iraurgi I. (2016). Desarrollo de una Escala de Autoinforme para la valoración del TDAH en la infancia (EA-TDAH): Estudio Delphi y datos de adecuación psicométrica. *Ter Psicol.* 34 41–52. 10.4067/S0718-48082016000100005 27315006

[B26] Sevincok D. Ozbay H. C. Ozbek M. M. Tunagur M. T. Aksu H. (2020). ADHD symptoms in relation to internalizing and externalizing symptoms in children: The mediating role of sluggish cognitive tempo. *Nord. J. Psychiat.* 74 265–272. 10.1080/08039488.2019.1697746 31809238

[B27] Tangney J. P. Baumeister R. F. Boone A. L. (2004). High self-control predicts good adjustment, less pathology, better grades, and interpersonal success. *J. Pers.* 72 271–324. 10.1111/j.0022-3506.2004.00263.x 15016066

[B28] Tsai C.-J. Chen Y.-L. Lin H.-Y. Gau S. S.-F. (2017). One-year trajectory analysis for ADHD symptoms and its associated factors in community-based children and adolescents in Taiwan. *Child. Adolesc. Psychiat. Ment. Health* 11:28. 10.1186/s13034-017-0165-4 28580012 PMC5452532

[B29] Umar M. U. Obindo J. T. Omigbodun O. O. (2018). Prevalence and Correlates of ADHD Among Adolescent Students in Nigeria. *J. Atten. Disord.* 22 116–126. 10.1177/1087054715594456 26220786

[B30] Vicente-Escudero J. L. Saura-Garre P. López-Soler C. Martínez A. Alcántara M. (2019). Adicción al móvil e internet en adolescentes y su relación con problemas psicopatológicos y variables protectoras. *Escr. Psicol.* 12 103–112. 10.24310/espsiescpsi.v12i2.10065

